# Treatment of Trachoma

**Published:** 1892-05-21

**Authors:** 


### OPHTHALMOLOGY.

### TREATMENT OF TRACHOMA.

In a recent communication, Dr. Kenneth Scott, of Cairo, gave the members of the Ophthalmological Society of the United Kingdom his experience of the treatment of trachoma, so very prevalent a disease in Egypt, by means of solutions of the perchloride of mercury, which he asserts is an "unfailingly effectual and rapid method." He makes an application of a 4 p.c. solution of perchloride of mercury in glycerine and water to the everted eyelid; this he brushes on every day, after applying cocain, and, in addition, gives the patient a solution of the same salt, only much weaker (¼ per cent.), to instil into the eyes three times a day. An iron tonic is also prescribed. Under such treatment a cure may be perfected in six or eight weeks, or usually much sooner.

He has had opportunities of watching the process of healing under this method of treatment, and finds the granulations first begin to decrease in size, gradually diminish in number, and ultimately disappear, leaving a smooth, polished surface, red and injected. Ultimately, the injection disappears, and only a thickening of the mucous membrane of the lids remains.

A 4 per cent. solution is a fairly powerful one to apply, but he has often found it is quite necessary, and that many cases which fail to improve under a weaker solution will yield to the stronger, though, at the same time, a 2 per cent. solution is often sufficiently strong.

Mr. Stephenson has largely used a 1 per cent. solution for the treatment of granular lids, and found it cure if sufficiently persevered with, a daily application for eighteen months, two years, or even longer. Trachoma first makes its appearance in upper and lower cul de sacs, formed by the junction of the eyelids with the globe of the eye, and spreads to the lids. Aggregations of lymphoid cells are found beneath the mucous membrane, with connective tissue forming at their bases, and small branches of blood vessels running into them; follicles are formed, and the adjacent papillæ hypertrophy, causing the lid to assume a rough granular appearance. The epithelial cells near the surface undergo fatty degeneration, and, together with the serous exudation, are discharged. After the involution of the epithelium and formation of vascular papillæ, the follicles are replaced by a fibro-cellular structure, which causes the tendency to sclerosis of the deeper structures.

If the epithelium remain intact the parts generally recover their normal character in the course of time; but if not, cicatrices are formed where the ulceration has been.

In the first stage of an attack, poultices applied warm, as recommended by Von Graefe, promote suppuration and the discharge of impurities which cause the disease, and are thus beneficial; but stimulants and absorbents are distinctly the most serviceable.

Dr. Wolfe, of Glasgow, introduced the treatment by astringents. He everts the eyelids and scarifies the tissues, including those of the cul de sac, and with the fingers, squeezes out the aggregations of lymphoid cells. He then sponges the surfaces with warm water to encourage bleeding, and applies atropin solution, followed by a lotion of borax three times a day. Two days later, syrup of tannin, according to his formula,* is poured on the everted eyelids, and the conjunctival surfaces are rubbed against one another. This treatment is repeated every two or three weeks, if necessary.

* By Acidi Tannici Pur. dm.ij., syrup simplicis cz.j. ♏. Heat in glass flask until a clear solution is formed.

